# The Small GTPase RhoA Localizes to the Nucleus and Is Activated by Net1 and DNA Damage Signals

**DOI:** 10.1371/journal.pone.0017380

**Published:** 2011-02-24

**Authors:** Adi D. Dubash, Christophe Guilluy, Melissa C. Srougi, Etienne Boulter, Keith Burridge, Rafael García-Mata

**Affiliations:** 1 Department of Cell and Developmental Biology, University of North Carolina at Chapel Hill, Chapel Hill, North Carolina, United States of America; 2 Lineberger Comprehensive Cancer Center, University of North Carolina at Chapel Hill, Chapel Hill, North Carolina, United States of America; University of Hong Kong, Hong Kong

## Abstract

**Background:**

Rho GTPases control many cellular processes, including cell survival, gene expression and migration. Rho proteins reside mainly in the cytosol and are targeted to the plasma membrane (PM) upon specific activation by guanine nucleotide exchange factors (GEFs). Accordingly, most GEFs are also cytosolic or associated with the PM. However, Net1, a RhoA-specific GEF predominantly localizes to the cell nucleus at steady-state. Nuclear localization for Net1 has been seen as a mechanism for sequestering the GEF away from RhoA, effectively rendering the protein inactive. However, considering the prominence of nuclear Net1 and the fact that a biological stimulus that promotes Net1 translocation out the nucleus to the cytosol has yet to be discovered, we hypothesized that Net1 might have a previously unidentified function in the nucleus of cells.

**Principal Findings:**

Using an affinity precipitation method to pulldown the active form of Rho GEFs from different cellular fractions, we show here that nuclear Net1 does in fact exist in an active form, contrary to previous expectations. We further demonstrate that a fraction of RhoA resides in the nucleus, and can also be found in a GTP-bound active form and that Net1 plays a role in the activation of nuclear RhoA. In addition, we show that ionizing radiation (IR) specifically promotes the activation of the nuclear pool of RhoA in a Net1-dependent manner, while the cytoplasmic activity remains unchanged. Surprisingly, irradiating isolated nuclei alone also increases nuclear RhoA activity via Net1, suggesting that all the signals required for IR-induced nuclear RhoA signaling are contained within the nucleus.

**Conclusions/Significance:**

These results demonstrate the existence of a functional Net1/RhoA signaling pathway within the nucleus of the cell and implicate them in the DNA damage response.

## Introduction

Rho GTPases are a family of proteins which control many different biological processes in the cell, including cell survival, proliferation, adhesion, migration, gene expression and apoptosis [1]. The Rho family of proteins contains at least 20 members, with RhoA, Rac1 and Cdc42 being among the best characterized [1]. These proteins function as molecular switches, cycling between an active GTP-bound form, and an inactive form that is bound to GDP [2]. The activation state of GTPases is regulated by three types of regulatory proteins: GEFs activate Rho proteins by catalyzing the exchange of GDP for GTP [3]; GTPase activating proteins (GAPs) inactivate them by promoting the intrinsic hydrolytic activity of the proteins [4]; finally, guanine nucleotide dissociation inhibitors (GDIs) bind to the GTPases and sequester them within the cytosol in an inactive conformation [5].

Subcellular localization of GTPases has also been identified as an important factor in the ability of GTPases to function in different signaling pathways [6]. Rho GTPases are primarily cytosolic proteins which associate with the PM via a C-terminal prenyl group (farnesyl or geranylgeranyl), which is added postranslationally to a C-terminal cysteine residue at the carboxy-terminal CAAX motif. Prenylation of GTPases allows for PM association and interaction with downstream effector proteins [7]. GDIs function to negatively regulate Rho proteins by extracting GTP-bound GTPases from the PM, and sequestering them in the cytosol [5]. Similarly, most Rho-GEFs localize either to the cytoplasm or to the PM [3]. However, at least two RhoA-specific GEFs, Net1 and Ect2, have been shown to localize preferentially within the nucleus at steady state [8], [9]. Both Net1 and Ect2 encode nuclear localization signals (NLS) that are required for their targeting to the nucleus [8], [9], [10], [11]. Deletion of the nuclear localization signals in Net1 promotes its redistribution to the cytoplasm, with the consequent activation of RhoA and the formation of stress fibers [8], [9], [10], [11].

Even though many studies have suggested important biological roles for Net1 and Ect2, it is unclear why both these GEFs are predominantly localized to the nucleus. Since the majority of RhoA is localized at the PM and in the cytosol of cells, the prevailing dogma in the field of Rho signaling has been that localization of Net1 to the nucleus is a mechanism designed to sequester it away from RhoA, therefore rendering nuclear Net1 biologically inert [9]. This is supported by data showing that a mutant of Net1 which is primarily cytosolic (lacking two of its NLS) causes cellular transformation, presumably as a result of upregulated RhoA signaling [10], [11]. A logical prediction of this hypothesis is that in order for Net1 to be functionally active, it must be transported out of the nucleus into the cytosol, where it can activate RhoA. However, a biological stimulus that causes translocation of Net1 from the nucleus to the cytosol has not yet been discovered. Considering the abundance of nuclear-localized Net1, we hypothesized that the nuclear pool of Net1 might serve a previously unidentified function regulating RhoA at this site.

In this study, we show that the majority of nuclear Net1 is in fact active. We also demonstrate that a fraction of the total RhoA pool localizes to the nucleus at steady state, and its activity is controlled by Net1. In addition, DNA damage signals such as ionizing radiation (IR), which has been previously shown to stimulate RhoA, specifically promoted the activation of the nuclear pool of RhoA in a Net1-dependent manner while the cytoplasmic activity was not affected. This IR-mediated increase in Net1 and RhoA activity occurred in isolated nuclei, suggesting the signals downstream of IR originated within the nucleus. These results represent the first demonstration that RhoA is present and can be activated in the nucleus by a Rho GEF in response to a specific stimulus such as IR.

## Results

### The RhoA-specific GEF Net1 is active in the nucleus of cells

**Figure 1:**
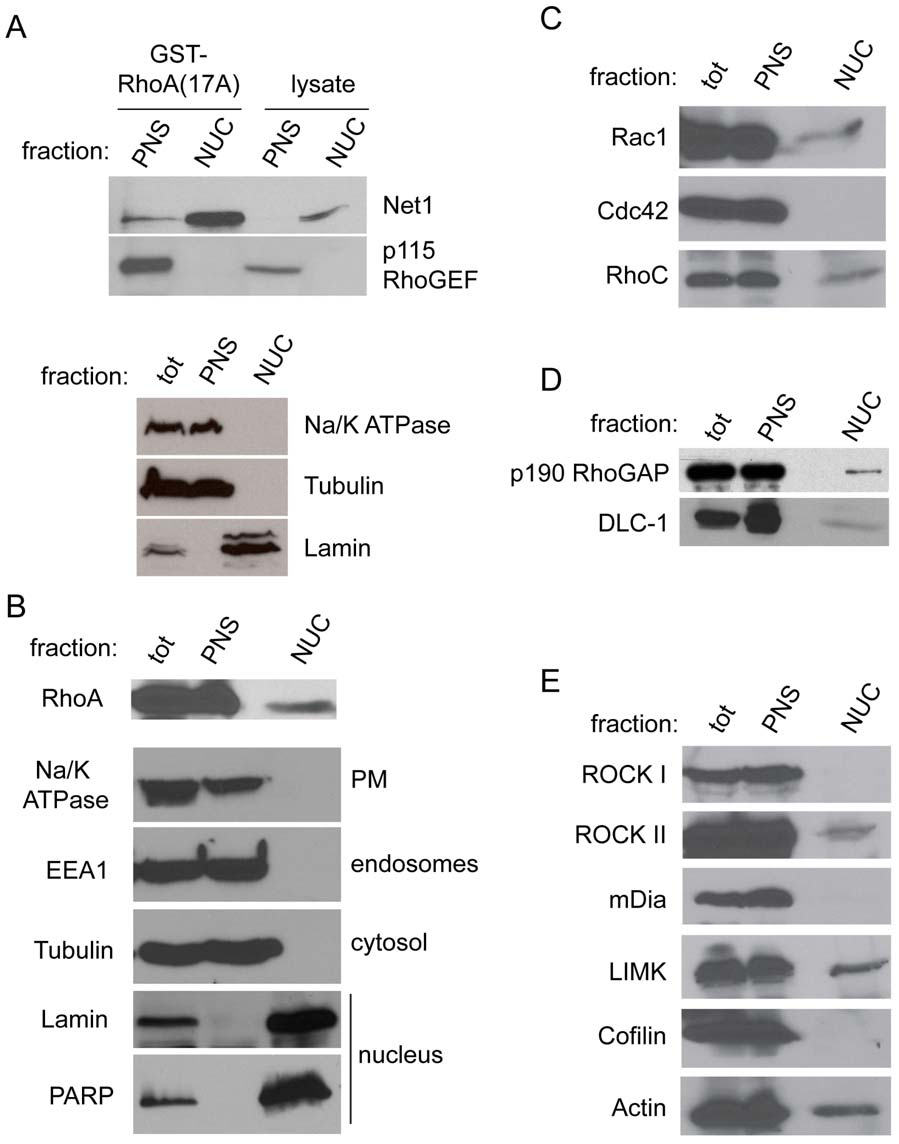
The RhoA exchange factor Net1 is active in the nucleus. (**A**) Active GEF pulldowns with GST-RhoA(17A) were performed from PNS and nuclear fractions of HEK293 cells, and the samples blotted with antibodies for the GEFs Net1 and p115 RhoGEF. (**B**) Samples were blotted for Na/K ATPase (plasma membrane), Tubulin (cytosol), and Lamin to monitor the purity of the nuclear fractions (Bottom panel). (**B–E**) Total cell lysate, PNS and nuclear fractions from HEK cells were immunoblotted for RhoA (**B**) to determine the amount of RhoA in the nucleus of cells, as well as other GTPases (**C**). The purity of the nuclear fractions was verified by blotting for different marker proteins, including Na/K ATPase (plasma membrane), Tubulin (cytosol), EEA1 (endosomes), and Lamin and PARP (nuclear) (**B**). Fractions were also blotted for a wide range of RhoA-related signaling proteins including GAPs (**D**), effectors and downstream signaling proteins (**E**) to determine their presence or absence in the nucleus.

To determine if Net1 might be active in the nucleus we used an assay previously developed in our laboratory to specifically precipitate the active pool of GEFs from cell lysates [12]. This assay takes advantage of a nucleotide-free single amino acid mutation in RhoA (G17A) that binds with high affinity to active RhoA-specific GEFs [12]. Since this assay had never been performed in nuclear lysates, we optimized a nuclear isolation protocol with emphasis on speed and purity of the isolated fractions (see Methods). The speed of the fractionation is critical since the efficiency of the assay decreases proportionally with time. The purity of our isolated nuclear fractions was routinely tested by blotting with different proteins commonly used as markers for various cellular fractions. PM (Na^+^/K^+^ ATPase), endosomal (EEA1), and cytosolic (tubulin) markers were observed in the total lysate and post nuclear supernatant (PNS), which includes the cytosolic and membrane fractions. Importantly, however, none of these contaminants were observed in the nuclear fraction within the detectable limit of these marker antibodies (Figure 1B).

Our results show that, as previously described [8], [9], endogenous Net1 is highly enriched in the nucleus, whereas other GEFs, such as p115 RhoGEF, are restricted to the PNS fraction (Figure 1A) [13]. Importantly, a significant amount of nuclear Net1 was precipitated with nucleotide-free RhoA, indicating that Net1 is indeed in an active form in the nucleus (Figure 1A). We estimate that approximately 25% of all nuclear Net1 is active at steady state. Another RhoA GEF, Ect2, is also precipitated with nucleotide-free RhoA from the nuclear fraction, suggesting that Ect2 is also present in an active form in the nucleus (Figure S1). To our knowledge, this is the first demonstration of the Rho GEFs Net1 and Ect2 being present in an active form in the nucleus of cells.

### Endogenous RhoA and multiple downstream signaling partners are present in the nucleus of cells

We next investigated whether RhoA was also present in the nucleus. While endogenous RhoA is predominantly localized to the cytoplasm of HEK cells, a low amount of endogenous RhoA is consistently detected in the nuclear fraction (Figure 1B). We estimate that approximately 5% of total cellular RhoA is localized to the nucleus of HEK cells at steady state. We have also tested other cell lines, including HeLa, with similar results (not shown).

In addition to RhoA, the closely related RhoC, as well as Rac1 were also detected in the nucleus (Figure 1C). In contrast, Cdc42 was not detected in the nucleus even after long exposures (Figure 1C). We also examined whether other upstream and downstream components in the Rho signaling pathway, such as RhoGAPs and effectors, were also present in the nucleus. While some of the proteins involved in RhoA signaling pathways, including the RhoA-GAP DLC1, p190 RhoGAP and the RhoA effectors ROCK II/ROKα and LIMK, were detected in the nucleus, others were not detected within the sensitivity of the assay, (ROCK I/ROKβ, mDia1, cofilin) (Figure 1D–E). We were also able to detect actin in the nucleus, as has been reported before by several groups (Figure 1E) [14]. From these data it is evident that a subset of RhoA-associated proteins are present in the nucleus, ranging from GEFs (Net1, Ect2), GAPs (DLC1, p190 RhoGAP) and GTPases (RhoA, RhoC, Rac1), to downstream effector proteins (ROCK II, LIMK). These results therefore highlight the potential of a functional nuclear Rho GTPase signaling cascade.

### RhoGDI1-binding restricts nuclear localization of RhoA

**Figure 2:**
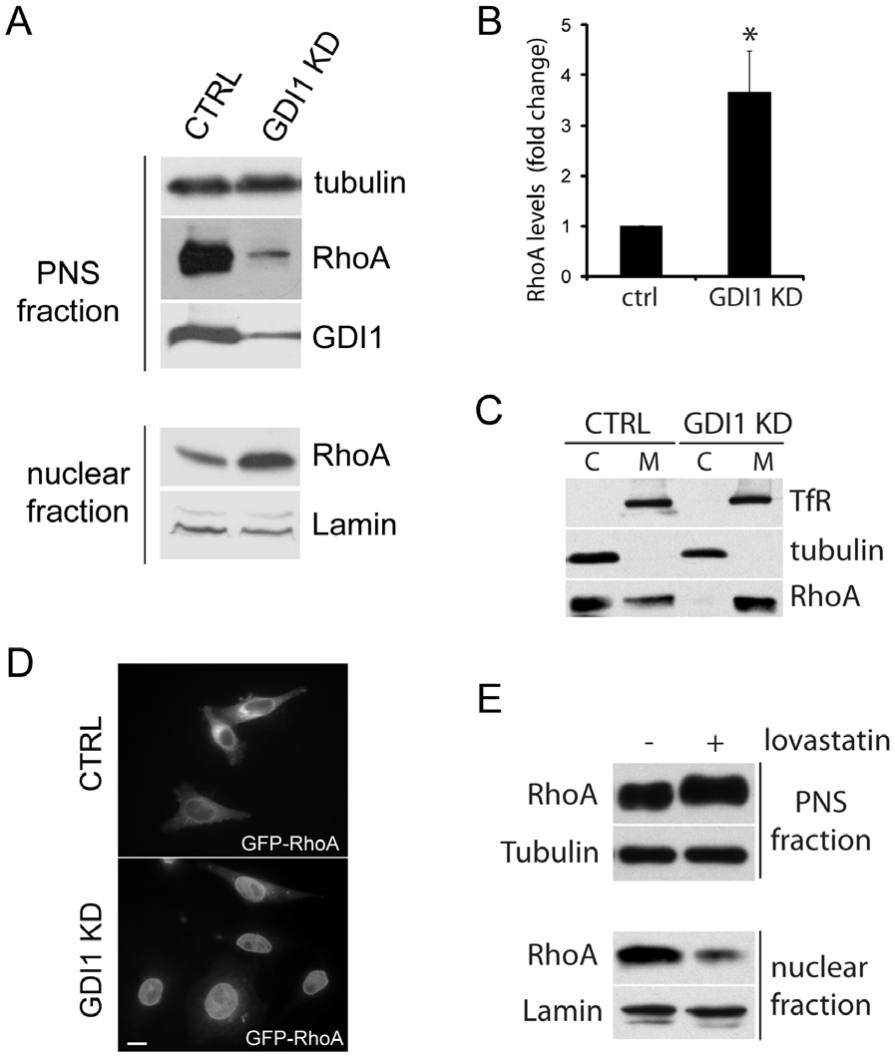
RhoGDI1 restricts nuclear localization of RhoA. (**A**) HEK293 cells were transfected with non-targeting control or Rho GDI1 siRNA oligonucleotides. 72 hours post-transfection, nuclei were isolated and lysates blotted for RhoA, GDI1, tubulin and Lamin. (**B**) Quantification of nuclear RhoA levels from three independent experiments is shown in the bar graph, as fold change over control cells. Asterisk indicate P = 0.03. (**C**) Control or RhoGDI1 siRNA-transfected cells, fractionated into cytosol (C) and membrane fractions (M), and blotted for RhoA, tubulin (cytosolic marker) and transferrin receptor (TfR, membrane marker). (**D**) HeLa cells were transfected with non-targeting control or Rho GDI1 siRNA oligonucleotides. 48 hours after transfection, cells were transfected with GFP-RhoA. 24 hours later, cells were fixed and imaged for localization of GFP-RhoA. Bar = 10 µm. (**E**) HEK cells were treated with 2.5 nM lovastatin for 48 h. PNS and nuclear fractions collected as detailed above, and samples blotted for RhoA, Lamin and Tubulin.

Previous studies have shown that the polybasic region (PBR) of Rac1 is important for its nuclear localization [15], [16]. The PBR of RhoA interacts strongly with RhoGDI, an association that keeps RhoA sequestered in the cytosol of cells and away from membranes where it is active [5]. To determine whether the interaction with Rho GDI might regulate the levels of RhoA in the nucleus, we used siRNA to knockdown Rho GDI1 expression. As we have recently described, RhoA protein level is drastically reduced upon RhoGDI1 silencing (Figure 2A) [17]. However, the amount of RhoA in the nuclear fraction increased significantly despite the overall reduction in RhoA levels (Figure 2A–B). As previously shown [17], the remaining pool of extranuclear RhoA is mostly associated with membranes (Figure 2C). A similar result is obtained when RhoGDI1 is silenced in cells that express GFP-RhoA. Figure 2D shows that, in control cells the majority of GFP-RhoA is diffusely distributed in the cytoplasm and regions surrounding the nucleus. However, after RhoGDI1 knockdown, the RhoA-GFP signal in the nucleus increases, with a concomitant reduction of fluorescence in the cytoplasm. This result suggests that RhoGDI1 may restrict the amount of RhoA that can be targeted to the nucleus.

It has been previously shown that inhibition of prenylation by statins or geranylgeranyl transferase inhibitors stabilizes the Rho GTPases in the cytosol, resulting in an increase of their expression levels [17], [18]. In contrast to the results obtained with RhoGDI1 silencing, where RhoA is still prenylated and targeting to the nucleus increased, inhibition of prenylation by lovastatin, showed a decrease in the levels of nuclear RhoA suggesting that only prenylated RhoA can be targeted efficiently to the nucleus (Figure 2E).

### Nuclear RhoA is in a GTP-bound active form, and is regulated by Net1

**Figure 3:**
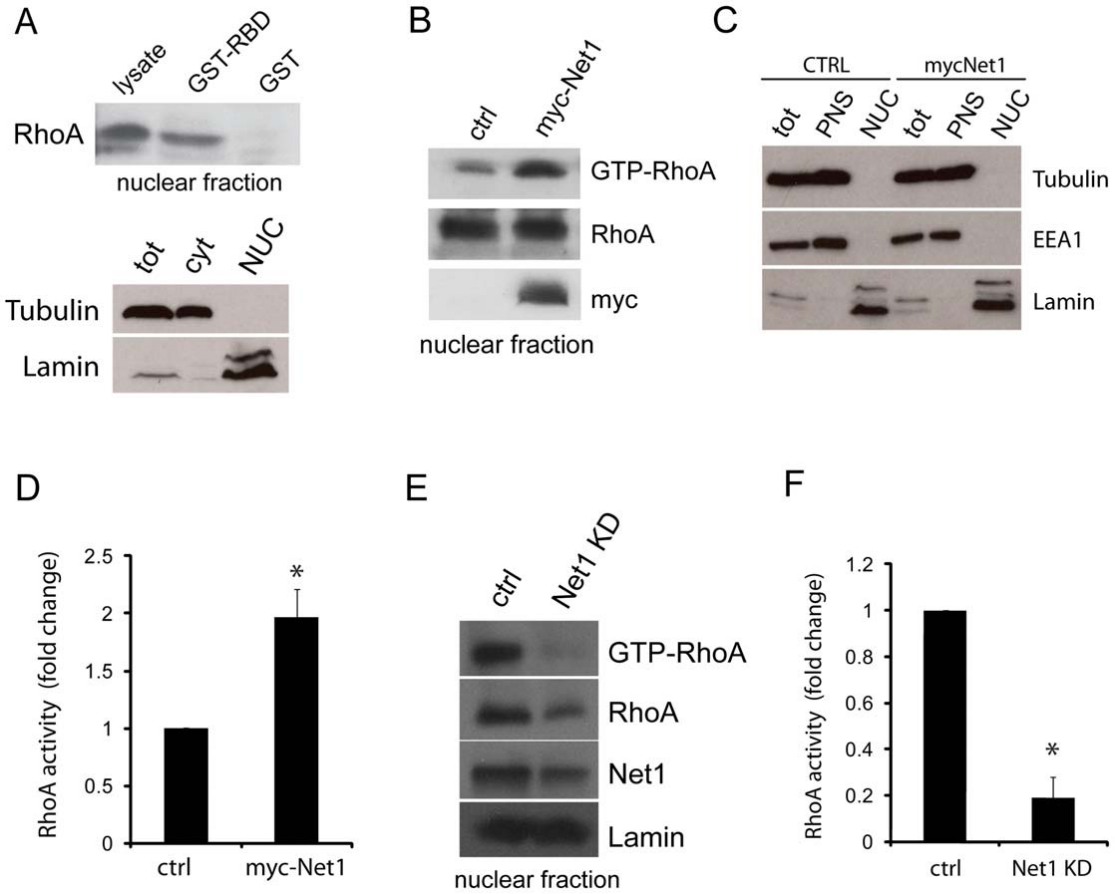
RhoA is active in the nucleus, and is regulated by Net1. (**A**) Nuclear lysates were incubated with GST-RBD (or GST alone as a control) to precipitate active RhoA from the nucleus of HEK293 cells. Samples were then subjected to SDS-PAGE and blotted with an anti-RhoA antibody. Samples were also blotted for Tubulin (cytosol), and Lamin to monitor the purity of the nuclear fractions (Bottom panel). (**B**) HEK cells were transfected with wildtype myc-tagged Net1 or a control vector. 24 hours after transfection, nuclear lysates were isolated and GST-RBD pulldowns performed. Samples were blotted for RhoA and exogenously expressed myc-Net1 (using an anti-myc antibody). (**C**) Samples in (B) were blotted for Tubulin (cytosol), EEA1(endosome) and Lamin (nucleus) to monitor the purity of the nuclear fractions. (**D**) Quantification of nuclear RhoA activity from three independent experiments is shown in the bar graph, as fold change over control cells. Asterisk indicate P = 0.0001 (n = 4). (**E**) HEK293 cells were transfected with control siRNA or Net1-specific siRNA. 72 hours post transfection, nuclear lysates were isolated and GST-RBD pulldowns performed. Samples were blotted for RhoA, Net1 and Lamin. (**F**) Quantification of nuclear RhoA activity from three independent experiments is shown in the bar graph, as fold change over control cells. Asterisk indicate P = 0.007 (n = 3).

We next wanted to determine if RhoA can exist in the nucleus in a biologically active GTP-bound form. To do this, we developed an assay to measure Rho-GTP levels from nuclear lysates based on the Rho-binding domain (RBD) pulldown assays [19]. As seen in Figure 3A, some nuclear RhoA is indeed in an active GTP-bound form at steady state. To determine if Net1 regulates nuclear RhoA, we first measured RhoA activity following overexpression of wild-type myc-tagged Net1. Our results show a significant increase in the nuclear activity of RhoA in Net1 overexpressing cells suggesting Net1 can modulate the activity of the nuclear pool of RhoA (Figure 3B–D). Supporting these results, silencing Net1 expression using siRNA oligonucleotides induces a significant reduction in nuclear RhoA activity (Figure 3E–F). These data indicate that the activity of nuclear RhoA can be regulated by Net1.

### IR treatment specifically increases activity of nuclear-localized RhoA and Net1

We next wanted to investigate the biological importance of Net1 and RhoA signaling in the nucleus of cells. Previous studies have shown that total Net1 activity is upregulated in response to extracellular stimuli that cause DNA damage, such as IR [20]. Correspondingly, RhoA has also been shown to be activated in response to DNA damaging agents [20], [21]. However, both of these studies investigated the activation of Net1 and RhoA from total cellular pools. While it was hypothesized that active Net1 translocates from the nucleus to activate RhoA in the cytosol, this has not been specifically demonstrated.

**Figure 4:**
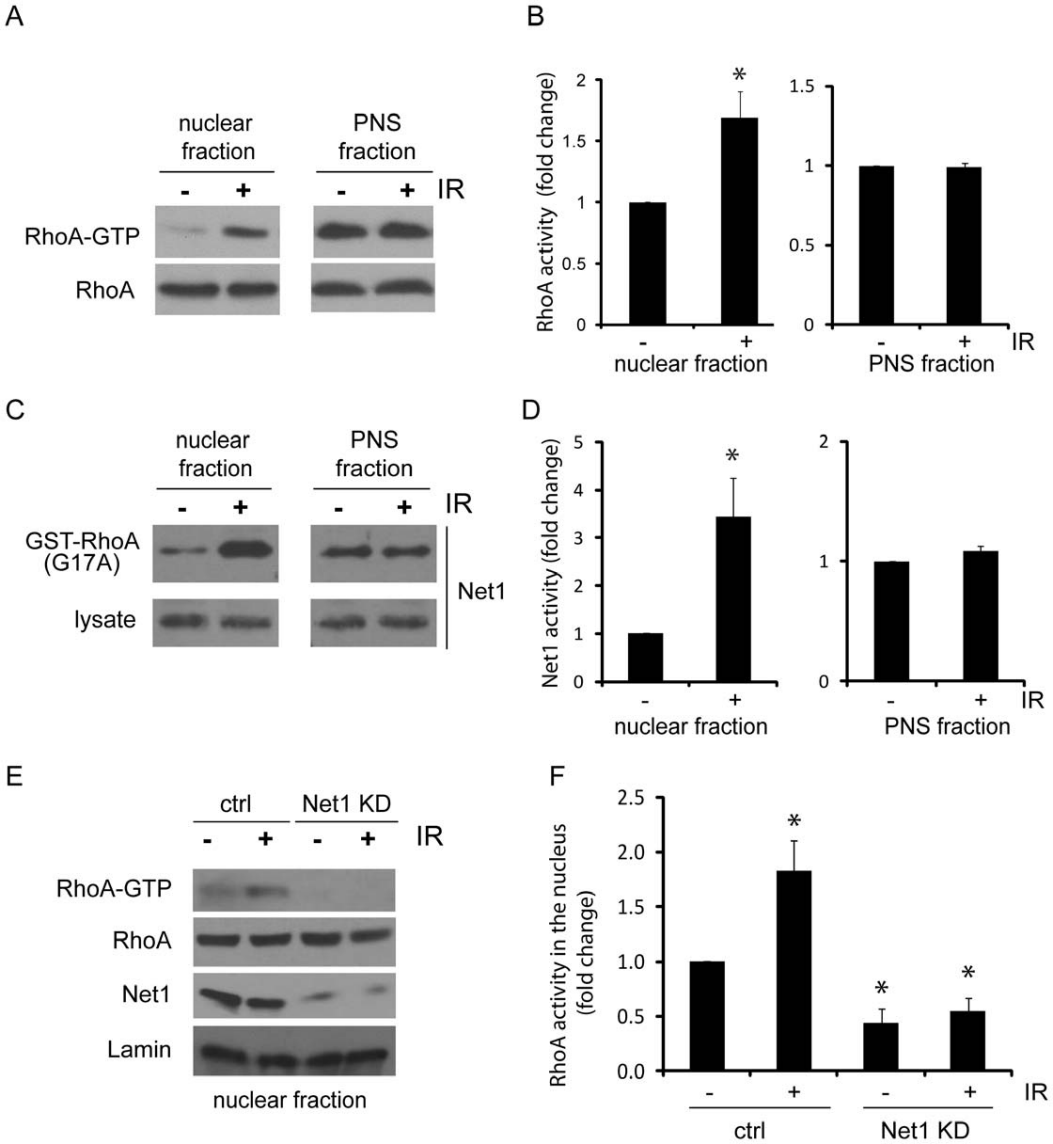
IR causes an increase in nuclear RhoA activity through Net1. HEK293 cells were either left untreated or exposed to ionizing radiation (10 Gy). After incubation at 37°C for 1 hr, PNS and nuclear fractions were isolated, and (**A**) RhoA and (**C**) GEF activity assays performed. The samples were then blotted with antibodies for the indicated proteins. (**E**) HEK293 cells were transfected with control siRNA or Net1-specific siRNA. 72 hours post transfection, the cells were either left untreated or exposed to ionizing radiation (10 Gy). After incubation at 37°C for 1 hr, nuclear fractions were isolated and Rho activity assays performed. Samples were subjected to SDS-PAGE and blotted with an anti-RhoA antibody. Samples were also blotted with anti-Net1 and anti-Lamin antibodies to demonstrate the level of Net1 knockdown. (**B, D and F**) Quantification of nuclear RhoA and Net1 activity from at least three independent experiments (n = 3) are shown in the bar graphs, as fold change over control cells. Asterisks in (B) indicate P = 0.01 (n = 6); in (D): P = 0.04 (n = 3); in (F): P = 0.02, P = 0.005 and P = 0.0125 respectively (n = 4).

Since we observed that Net1 and RhoA are both active in the nucleus, we wanted to determine whether the previously observed IR-induced increase in RhoA and Net1 activity occurred in the nucleus or in the cytoplasm. To do this, HEKs were either left untreated, or irradiated, and RhoA activity assays performed from isolated PNS and nuclear fractions. Interestingly, while the activity of nuclear-localized RhoA increased significantly in response to IR exposure, the activity of RhoA in the cytoplasm (PNS fraction) was not affected (Figure 4A–B). In support of this data, we see no major changes in the amount of stress fiber formation in response to irradiation (Figure S2, A–B). The specific activation of nuclear RhoA suggests that this subcellular pool of RhoA is important for downstream activation of DNA damage signaling.

Similar to nuclear RhoA, the activity of nuclear Net1 was also upregulated by IR while the activity in the cytoplasm (PNS fraction) remained unchanged (Figure 4C–D). To determine if Net1 is involved in the IR-induced increase in nuclear RhoA activity, we silenced Net1 expression using siRNA oligonucleotides. Net1 knockdown cells displayed lower basal levels of active RhoA in the nucleus than control cells, and failed to activate the nuclear RhoA fraction in response to IR (Figure 4E–F). In contrast to the results observed in nuclear RhoA activity, simultaneous analysis of RhoA activity in the PNS fraction showed no significant effect by Net1 silencing in either untreated or irradiated cells (Figure S2C). These data therefore suggest that Net1 is the major GEF responsible for activating nuclear RhoA in response to a DNA damage stimulus.

**Figure 5:**
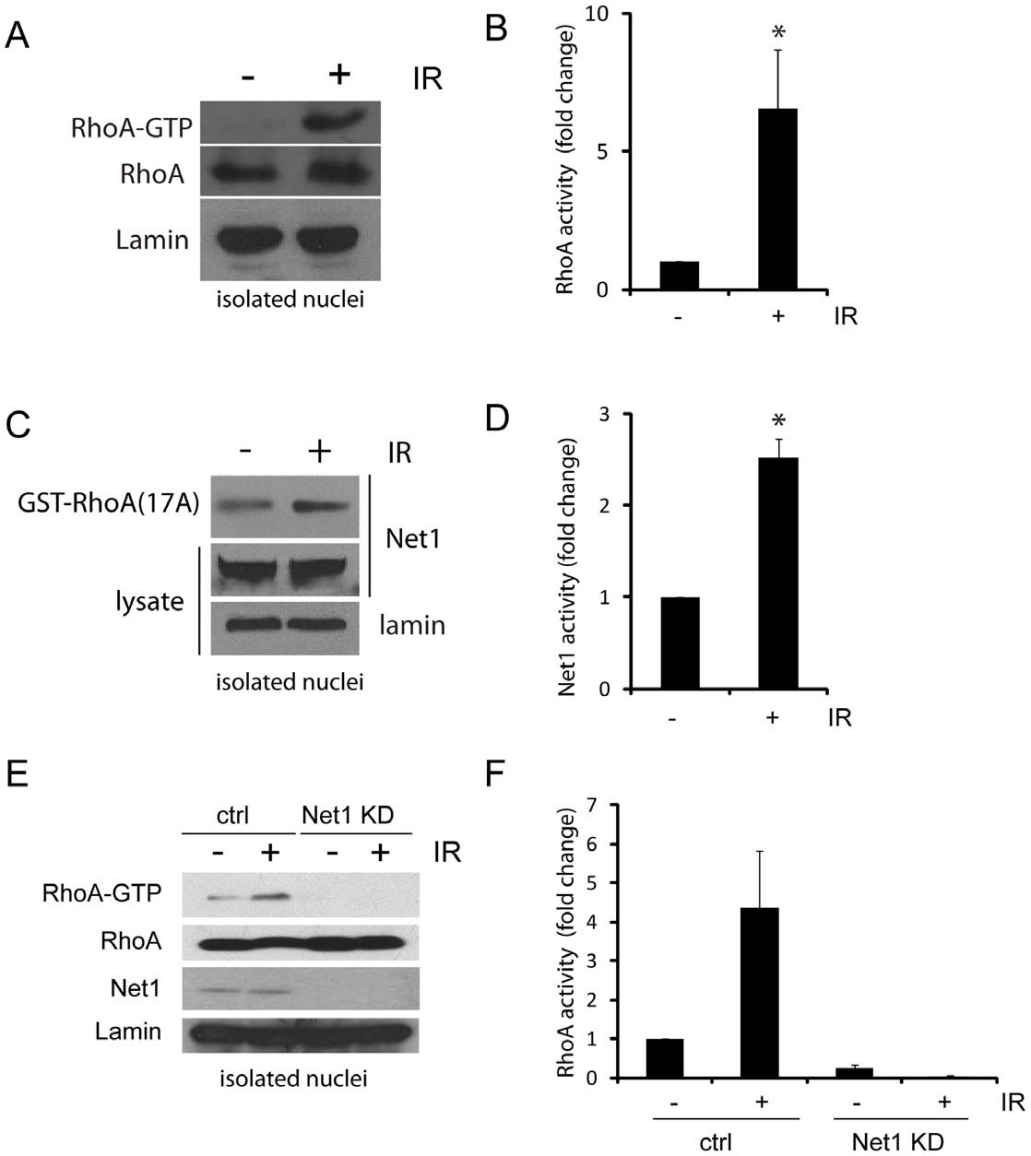
IR promotes Net1-mediated RhoA activation in isolated nuclei. Nuclei were isolated from HEK293 cells, and either left untreated or exposed to ionizing radiation (10 Gy). After IR, nuclei were immediately processed for (**A**) RhoA activity or (**C**) GEF activity assays, and samples blotted for the indicated proteins. (**E**) HEK293 cells were transfected with control siRNA or Net1-specific siRNA. 72 hours post transfection; nuclei were isolated, irradiated, processed for RhoA activity assays, and blotted with antibodies to Net1, RhoA and Lamin. (**B, D and F**) Quantification of nuclear RhoA and Net1 activity in isolated nuclei are shown in the bar graphs, as fold change over control cells. Asterisk in (B) and (D) indicate P = 0.04 (n = 4) and P = 0.001 (n = 3) respectively; n = 2 in (F).

Our experimental conditions do not exclude the possibility that RhoA and Net1 are activated in the cytoplasm and shuttled into the nucleus during the assay. To address this issue, we first isolated nuclear fractions from HEK cells, subjected the isolated nuclei to IR, and then performed RhoA and Net1 activity assays. Our results show that both RhoA and Net1 can be activated by IR in isolated nuclei in the absence of any signals from the cytoplasm (Figure 5A–D). In addition, isolated nuclei failed to activate RhoA following IR when Net1 expression was silenced (Figure 5E–F). These results suggest that the components required for activation of Net1 following IR are all present in the nucleus. In summary, these results highlight a previously unappreciated role for Net1 activity in the nucleus of cells, and implicate Net1-RhoA nuclear signaling in the biological response to DNA damage.

## Discussion

Net1 and Ect2 are unique among RhoA GEFs in their predominant localization to nuclei at steady-state. Another RhoA GEF, XPLN, has also been seen in the nucleus, but this was only determined by overexpression of a GFP-tagged XPLN construct [22]. The functions of Net1 and Ect2 have been tied to different biological processes: Net1 has been shown to be involved in regulation of transforming growth factor-β and serum response factor signaling [10], [23], and Ect2 has been implicated in the regulation of RhoA and Cdc42 in different cell cycle-related processes [24], [25], [26]. However, no previous study has determined the importance of nuclear localization of Net1, or if nuclear Net1 has a biological function. In this study we show that a significant amount of Net1 is indeed active in the nucleus of different cell types (Figure 1), contrary to studies that assumed nuclear Net1 was biologically inactive.

In addition, we demonstrate that a small fraction of cellular RhoA is present in the nucleus. Importantly, RhoA can be detected in the nucleus in its GTP-bound active form, and its activation state is regulated by Net1 (Figures 2 and 3). Other GTPases are also present in the nucleus, like Rac1 and RhoC. These data are supported by a previous report describing the presence of Rac1, and to a lesser extent RhoA, in the nucleus [16]. Further, different RhoA signaling partners also localize to the nucleus, including GAPs (DLC1, p190 RhoGAP) and effector proteins (ROCK II, LIMK). Our data is supported by previous reports describing the nuclear localization of these proteins [27], [28], [29]. Activity of ROCK II in the nucleus has been shown to regulate the phosphorylation and activation of p300 acetyltransferase [28]. ROCK phosphorylates and activates LIMK, which has also been reported to be nuclear-localized [29]. While we did not detect cofilin in the nucleus of HEK cells, it has been reported in the nucleus by several groups [30], [31]. However, cofilin seems to accumulate in the nucleus only in cells exposed to specific stress stimuli (such as ATP depletion or heat shock), suggesting that cofilin may not normally have a nuclear localization in healthy cells at steady state [30], [31]. Not surprisingly, actin was also detected in the nucleus, which has been previously implicated in several different nuclear processes, such as transcription and chromatin remodeling [14]. Interestingly, focal adhesion proteins like zyxin and paxillin have also been found in the nucleus, where they have been implicated in regulating the transcriptional activities of specific genes [32], [33], [34]. Cumulatively, these data highlight the presence of major Rho-related signaling processes in the nucleus of cells, an area that has previously been neglected.

Previous studies have shown that the PBR of Rac1 and several adjoining residues comprise a functional NLS that is responsible for the nuclear localization of Rac1 [15], [16]. Expression of constructs expressing the PBRs of both Rac1 and RhoA tagged to GFP showed that the PBRs of both proteins functioned as NLSs, with the Rac1 PBR being a stronger NLS [35]. The PBR of RhoA has been shown to strongly interact with Rho GDI1, an association that keeps RhoA sequestered in the cytoplasm of cells. Our results show that RhoA levels in the nucleus increase upon knockdown of RhoGDI1, suggesting that binding to RhoGDI1 plays a role in modulating RhoA levels in the nucleus. Our results suggest that nuclear RhoA is prenylated in agreement with a previous report showing that nuclear Rac1 is also prenylated [16].

Previous studies have shown that Net1 is regulated via phosphorylation by PAK1, which inhibits its exchange activity [36]. IR was shown to cause a reduction in total phosphorylated Net1, which is indicative of GEF activation [20]. This study also showed that IR-induced Net1 activity was also responsible for downstream activation of the MAPK pathway, leading to an increase in cell survival [20]. An assumption of these experiments was that active Net1 translocates from the nucleus to the cytosol, where it can interact with and activate RhoA [20]. However, we show here that IR specifically activates the nuclear pool of RhoA (not the cytosolic pool), in addition to nuclear Net1 (Figure 4). Importantly, Net1 is required for IR-induced nuclear RhoA activity. These data demonstrate a novel role for Net1 in the nucleus of cells, and implicate both nuclear Net1 and RhoA in DNA damage signaling.

We have recently shown that Net1 interacts with several members of the Dlg family of tumor suppressors via its PDZ-binding domain [37]. Interestingly, Net1 colocalizes with these proteins in discrete punctate nuclear structures that are associated with promyelocytic leukemia (PML) bodies. PML nuclear bodies are multiprotein complexes involved in regulating the cellular response to DNA damage [38]. Localization of Net1 to PML bodies is therefore consistent with this GEF contributing to the signaling cascades initiated by DNA damage.

We also confirm that the signals required for activation of Net1 (and RhoA) are generated in the nucleus, as isolated nuclei exposed to IR also show this effect (Figure 5). It is interesting to speculate how Net1 might be activated in the nucleus of cells responding to IR. Since phosphorylated Net1 is inactive [36], it is likely that activation of Net1 involves dephosphorylation by a phosphatase, which itself could be activated by several different kinases stimulated by IR. A particularly interesting candidate is Ataxia-telangiectasia mutated (ATM), a protein that plays a major role in regulating the cellular response to DNA damage [21]. IR triggers a rapid activation of ATM, which is responsible for phosphorylating several different downstream targets involved in cell cycle arrest and DNA repair [39], [40]. Importantly, RhoA activation in response to DNA damage was shown to be inhibited in ATM deficient cells [21], suggesting that ATM may be involved in the stimulation of Net1 by IR. Our future work will focus on investigating the mechanisms by which Net1 is activated in response to IR.

## Materials and Methods

### Cell Culture and Reagents

HEK293 and HeLa cells were cultured in Dulbecco's modified Eagle's medium (DMEM) (Invitrogen) supplemented with 10% fetal bovine serum (FBS) (Sigma), and antibiotics (penicillin-streptomycin). For inhibition of prenylation, HEK cells were treated overnight with 2.5 nM lovastatin (Axxora).

### Subcellular fractionation

To isolate pure nuclear fractions from whole cell lysates, cells were first washed with Tris-buffered saline with 1 mM MgCl_2_, and scraped in a hypotonic buffer (10 mM pH 7.9 Hepes, 1.5 mM MgCl_2_, 10 mM KCl, 0.5 mM DTT plus protease inhibitors). After incubating 5 minutes on ice, samples are homogenized using 20 strokes of a tight-fitting Dounce homogenizer. An aliquot is retained as the total cellular fraction. The homogenate was then centrifuged at 400× *g* for 3 min to produce a post nuclear supernatant fraction (PNS) and a crude nuclear fraction (pellet). The nuclear pellet was resuspended in a 30% iodixanol solution (Optiprep, Axis Shield), and centrifuged at 12,000× *g* for 4 min. This last centrifugation step was repeated a second time, and the pellet obtained was used as a pure nuclear fraction. The obtained total, PNS (cytoplasmic) and nuclear fractions were then processed for pulldown experiments or microscopy. For cytosol (C) and membrane (M) fractionation, the PNS fraction from the first low speed centrifugation in the nuclear isolation protocol described above was spun at 40,000× *g* for 30 min at 4°C and the pellets corresponding to the total membrane fraction, were gently washed once with hypotonic lysis buffer. Typically, 5% of the cytosolic fraction and 25% of the membrane fraction were analysed by SDS–PAGE and western blotting.

### RhoA and GEF activity assays

Construction of the GST-RBD and GST-RhoA(G17A) prokaryotic expression constructs and purification of the recombinant proteins has been described in detail elsewhere [41], [42]. Active RhoA pulldown assays and affinity precipitation of exchange factors with the nucleotide-free RhoA mutant (G17A) has been described in detail in previous work [12], [19]. For RhoA pulldowns, samples (total, PNS or nuclear fractions) were reconstituted in 50 mM Tris (pH 7.6), 500 mM NaCl, 1% Triton X-100, 0.1% SDS, 0.5% deoxycholate, 10 mM MgCl_2_, 200 µM orthovanadate plus protease inhibitors, and sonicated briefly. Samples were equalized for total protein concentrations, and rotated for 30 min at 4°C with 30–60 µg of purified GST-tagged Rhotekin Rho-binding domain (RBD) bound to glutathione-sepharose beads. The bead pellets were washed in 50 mM Tris (pH 7.6), 150 mM NaCl, 1% Triton X-100, 10 mM MgCl_2_, 200 µM orthovanadate, with protease inhibitors, and subsequently processed for SDS-PAGE.

For affinity precipitation of exchange factors, total, PNS or nuclear fractions were reconstituted in 20 mM Hepes (pH 7.6), 150 mM NaCl, 1% Triton X-100, 5 mM MgCl_2_, 200 µM orthovanadate plus protease inhibitors, and sonicated briefly. Lysates were equalized for protein concentration and incubated with 20 µg of purified GST-tagged RhoA(17A) bound to glutathione-sepharose beads for 60 min at 4°C. Samples were then washed with lysis buffer and processed for SDS-PAGE. For IR experiments, HEK cells were either left untreated or exposed to ionizing radiation (10 Gy), and then incubated at 37°C for 1 hr in 5% CO_2_. PNS and nuclear fractions were then isolated from these cells, followed by either RBD or GEF pulldowns. For IR of isolated nuclei, nuclear fractions from HEK cells were resuspended in 20 mM Tris-HCl, pH 7.8, 0.25 M Sucrose, 25 mM KCl, 5 mM MgCl_2_, 1 mM ATP, 1 mM GTP and exposed to IR (10 Gy). Following irradiation, nuclei were subjected to RhoA or GEF activity assays.

### Transfections and imaging

Transfection of HEK 293 and HeLa cells was performed using FuGene6 Reagent, according to manufacturer's instructions (Roche). To determine purity of nuclear fractions, small aliquots were resuspended in mounting medium containing 1 µg/ml Hoechst to stain nuclear DNA. For F-actin staining, HeLa cells were grown on coverslips, and subjected to irradiation (10 Gy). The irradiated cells were incubated at 37°C for various time points, and fixed and processed for immunofluoresence staining with Alexa 488-Phalloidin. Quantitative analysis of stress fibers was performed using Metamorph to measure the average fluorescence intensity per cell in images that were serially acquired using the same illumination and exposure parameters. Images were taken with a Zeiss axiovert 200 M microscope equipped with a Hamamatsu ORCA-ERAG digital camera and Metamorph Workstation (Molecular Devices).

### siRNA oligonucleotides

Control or targeted small interfering RNA (siRNA) oligonucleotides were purchased either from Dharmacon or the Nucleic Acids Core Facility at UNC-Chapel Hill. The sequences were as follows: control (5′-UCACUCGUGCCGCAUUUCCTT-3′), human Net1 (5′-GAGUCUCCCUUCAGUCGAA-3′), and human GDI1 (5′-UCAAUCUUGACGCCUUUCCTT-3′). Transfection of oligonucleotides was performed using TransIT-siQUEST (Mirus Corporation) according to manufacturer's instructions.

### Western blotting

The following primary antibodies were used: anti-Lamin A/C, anti-EEA1, anti-Rac1, anti-Cdc42, anti-ROCKI, anti-ROCKII, anti-mDia1 (BD Transduction Laboratories), mouse monoclonal anti-RhoA, anti-RhoC, anti-p115 RhoGEF, anti-Ect2, anti-GDI1 (Santa Cruz Biotechnology), anti-Cofilin, rabbit monoclonal anti-RhoA and anti-PARP (Cell Signaling Technologies), anti-Actin, anti-p190RhoGAP (Millipore), anti-Tubulin (Sigma), anti-Na/K ATPase (Abcam) and anti-myc (Invitrogen). DLC1 antibodies were a gift from Channing Der (UNC). A rabbit polyclonal antibody against Net1 was made through Pacific Immunologicals, using a peptide containing the last 18 amino acids of human Net1 (C-RRARDKALSGGKRKETLV). Quantification of all blots was performed by densitometry using Image J software (NIH), and represented in the bar graphs as fold increase over control cells from three independent experiments. Error bars represent S.E.M. Statistical differences between two groups of data were analysed with a two-tailed unpaired Student's *t*-test. To estimate the amount of RhoA and active Net1 in the nucleus of cells, band intensity of RhoA/Net1 in nuclear vs. total cellular fractions was measured by densitometry, and the total amounts were calculated based on the percentage of the total lysate loaded in the gel followed by active/total ratio calculations.

## Supporting Information

Figure S1 **The RhoA GEF Ect2 is active in the nucleus of cells.** Active GEF pulldowns with GST-RhoA(17A) were performed from PNS and nuclear fractions of HEK293 cells, and the samples blotted with antibodies for the GEF Ect2.
(TIF)

Figure S2 **IR does not affect cytosolic RhoA signaling.** (**A**) HeLa cells grown on coverslips were exposed to ionizing radiation (10 Gy), and fixed after incubation at 37°C for the indicated times. Cells were then stained with Alexa 488-Phalloidin to visualize F-actin. (**B**) Stress Fibers were quantified as described in Materials and Methods (n = 180 for each time point). Bar =  10µM. (**C**) HEK293 cells were transfected with control siRNA or Net1-specific siRNA. 72 hours post transfection, cells were either left untreated or exposed to ionizing radiation (10 Gy). After IR, cytosolic fractions were processed for RhoA activity assays and blotted for RhoA and Tubulin. This experiment was done simultaneously with the one shown in Figure 4E so the panel showing the efficiency of Net1 KD is shown there. Quantification of cytoplasmic (PNS) RhoA activity from three independent experiments (n = 3) is shown in the bar graph, as fold change over control cells.
(TIF)

## References

[1] Hall A (2005). Rho GTPases and the control of cell behaviour.. Biochem Soc Trans.

[2] Burridge K, Wennerberg K (2004). Rho and Rac take center stage.. Cell.

[3] Rossman KL, Der CJ, Sondek J (2005). GEF means go: turning on RHO GTPases with guanine nucleotide-exchange factors.. Nat Rev Mol Cell Biol.

[4] Moon SY, Zheng Y (2003). Rho GTPase-activating proteins in cell regulation.. Trends Cell Biol.

[5] DerMardirossian C, Bokoch GM (2005). GDIs: central regulatory molecules in Rho GTPase activation.. Trends Cell Biol.

[6] Mor A, Philips MR (2006). Compartmentalized Ras/MAPK signaling.. Annu Rev Immunol.

[7] Seabra MC (1998). Membrane association and targeting of prenylated Ras-like GTPases.. Cell Signal.

[8] Chalamalasetty RB, Hummer S, Nigg EA, Sillje HH (2006). Influence of human Ect2 depletion and overexpression on cleavage furrow formation and abscission.. J Cell Sci.

[9] Schmidt A, Hall A (2002). The Rho exchange factor Net1 is regulated by nuclear sequestration.. J Biol Chem.

[10] Alberts AS, Treisman R (1998). Activation of RhoA and SAPK/JNK signalling pathways by the RhoA-specific exchange factor mNET1.. Embo J.

[11] Chan AM, Takai S, Yamada K, Miki T (1996). Isolation of a novel oncogene, NET1, from neuroepithelioma cells by expression cDNA cloning.. Oncogene.

[12] Garcia-Mata R, Wennerberg K, Arthur WT, Noren NK, Ellerbroek SM (2006). Analysis of activated GAPs and GEFs in cell lysates.. Methods Enzymol.

[13] Dubash AD, Wennerberg K, Garcia-Mata R, Menold MM, Arthur WT (2007). A novel role for Lsc/p115 RhoGEF and LARG in regulating RhoA activity downstream of adhesion to fibronectin.. J Cell Sci.

[14] Pederson T (2008). As functional nuclear actin comes into view, is it globular, filamentous, or both?. J Cell Biol.

[15] Lanning CC, Daddona JL, Ruiz-Velasco R, Shafer SH, Williams CL (2004). The Rac1 C-terminal polybasic region regulates the nuclear localization and protein degradation of Rac1.. J Biol Chem.

[16] Michaelson D, Abidi W, Guardavaccaro D, Zhou M, Ahearn I (2008). Rac1 accumulates in the nucleus during the G2 phase of the cell cycle and promotes cell division.. J Cell Biol.

[17] Boulter E, Garcia-Mata R, Guilluy C, Dubash A, Rossi G (2010). Regulation of Rho GTPase crosstalk, degradation and activity by RhoGDI1.. Nat Cell Biol.

[18] Dunford JE, Rogers MJ, Ebetino FH, Phipps RJ, Coxon FP (2006). Inhibition of protein prenylation by bisphosphonates causes sustained activation of Rac, Cdc42, and Rho GTPases.. J Bone Miner Res.

[19] Ren XD, Kiosses WB, Schwartz MA (1999). Regulation of the small GTP-binding protein Rho by cell adhesion and the cytoskeleton.. Embo J.

[20] Guerra L, Carr HS, Richter-Dahlfors A, Masucci MG, Thelestam M (2008). A bacterial cytotoxin identifies the RhoA exchange factor Net1 as a key effector in the response to DNA damage.. PLoS ONE.

[21] Frisan T, Cortes-Bratti X, Chaves-Olarte E, Stenerlow B, Thelestam M (2003). The Haemophilus ducreyi cytolethal distending toxin induces DNA double-strand breaks and promotes ATM-dependent activation of RhoA.. Cell Microbiol.

[22] Arthur WT, Ellerbroek SM, Der CJ, Burridge K, Wennerberg K (2002). XPLN, a guanine nucleotide exchange factor for RhoA and RhoB, but not RhoC.. J Biol Chem.

[23] Shen X, Li J, Hu PP, Waddell D, Zhang J (2001). The activity of guanine exchange factor NET1 is essential for transforming growth factor-beta-mediated stress fiber formation.. J Biol Chem.

[24] Kamijo K, Ohara N, Abe M, Uchimura T, Hosoya H (2006). Dissecting the role of Rho-mediated signaling in contractile ring formation.. Mol Biol Cell.

[25] Oceguera-Yanez F, Kimura K, Yasuda S, Higashida C, Kitamura T (2005). Ect2 and MgcRacGAP regulate the activation and function of Cdc42 in mitosis.. J Cell Biol.

[26] Tatsumoto T, Xie X, Blumenthal R, Okamoto I, Miki T (1999). Human ECT2 is an exchange factor for Rho GTPases, phosphorylated in G2/M phases, and involved in cytokinesis.. J Cell Biol.

[27] Scholz RP, Regner J, Theil A, Erlmann P, Holeiter G (2009). DLC1 interacts with 14-3-3 proteins to inhibit RhoGAP activity and block nucleocytoplasmic shuttling.. J Cell Sci.

[28] Tanaka T, Nishimura D, Wu RC, Amano M, Iso T (2006). Nuclear Rho kinase, ROCK2, targets p300 acetyltransferase.. J Biol Chem.

[29] Yokoo T, Toyoshima H, Miura M, Wang Y, Iida KT (2003). p57Kip2 regulates actin dynamics by binding and translocating LIM-kinase 1 to the nucleus.. J Biol Chem.

[30] Nishida E, Iida K, Yonezawa N, Koyasu S, Yahara I (1987). Cofilin is a component of intranuclear and cytoplasmic actin rods induced in cultured cells.. Proc Natl Acad Sci U S A.

[31] Pendleton A, Pope B, Weeds A, Koffer A (2003). Latrunculin B or ATP depletion induces cofilin-dependent translocation of actin into nuclei of mast cells.. J Biol Chem.

[32] Hervy M, Hoffman L, Beckerle MC (2006). From the membrane to the nucleus and back again: bifunctional focal adhesion proteins.. Curr Opin Cell Biol.

[33] Kasai M, Guerrero-Santoro J, Friedman R, Leman ES, Getzenberg RH (2003). The Group 3 LIM domain protein paxillin potentiates androgen receptor transactivation in prostate cancer cell lines.. Cancer Res.

[34] Nix DA, Beckerle MC (1997). Nuclear-cytoplasmic shuttling of the focal contact protein, zyxin: a potential mechanism for communication between sites of cell adhesion and the nucleus.. J Cell Biol.

[35] Lanning CC, Ruiz-Velasco R, Williams CL (2003). Novel mechanism of the co-regulation of nuclear transport of SmgGDS and Rac1.. J Biol Chem.

[36] Alberts AS, Qin H, Carr HS, Frost JA (2005). PAK1 negatively regulates the activity of the Rho exchange factor NET1.. J Biol Chem.

[37] Garcia-Mata R, Dubash AD, Sharek L, Carr HS, Frost JA (2007). The nuclear RhoA-exchange factor Net1 interacts with proteins of the Dlg family, affects their localization and influences their tumor suppressor activity..

[38] Dellaire G, Bazett-Jones DP (2007). Beyond repair foci: subnuclear domains and the cellular response to DNA damage.. Cell Cycle.

[39] Bakkenist CJ, Kastan MB (2004). Initiating cellular stress responses.. Cell.

[40] Bentle MS, Bey EA, Dong Y, Reinicke KE, Boothman DA (2006). New tricks for old drugs: the anticarcinogenic potential of DNA repair inhibitors.. J Mol Histol.

[41] Liu BP, Burridge K (2000). Vav2 activates Rac1, Cdc42, and RhoA downstream from growth factor receptors but not beta1 integrins.. Mol Cell Biol.

[42] Reuther GW, Lambert QT, Booden MA, Wennerberg K, Becknell B (2001). Leukemia-associated Rho guanine nucleotide exchange factor, a Dbl family protein found mutated in leukemia, causes transformation by activation of RhoA.. J Biol Chem.

