# Supplementary figures and images for: The Small GTPase RhoA Localizes to the Nucleus and Is Activated by Net1 and DNA Damage Signals
Source: PLoS One. 2011 Feb 24;6(2):e17380. doi: 10.1371/journal.pone.0017380 (PMC3044755; doi:10.1371/journal.pone.0017380)

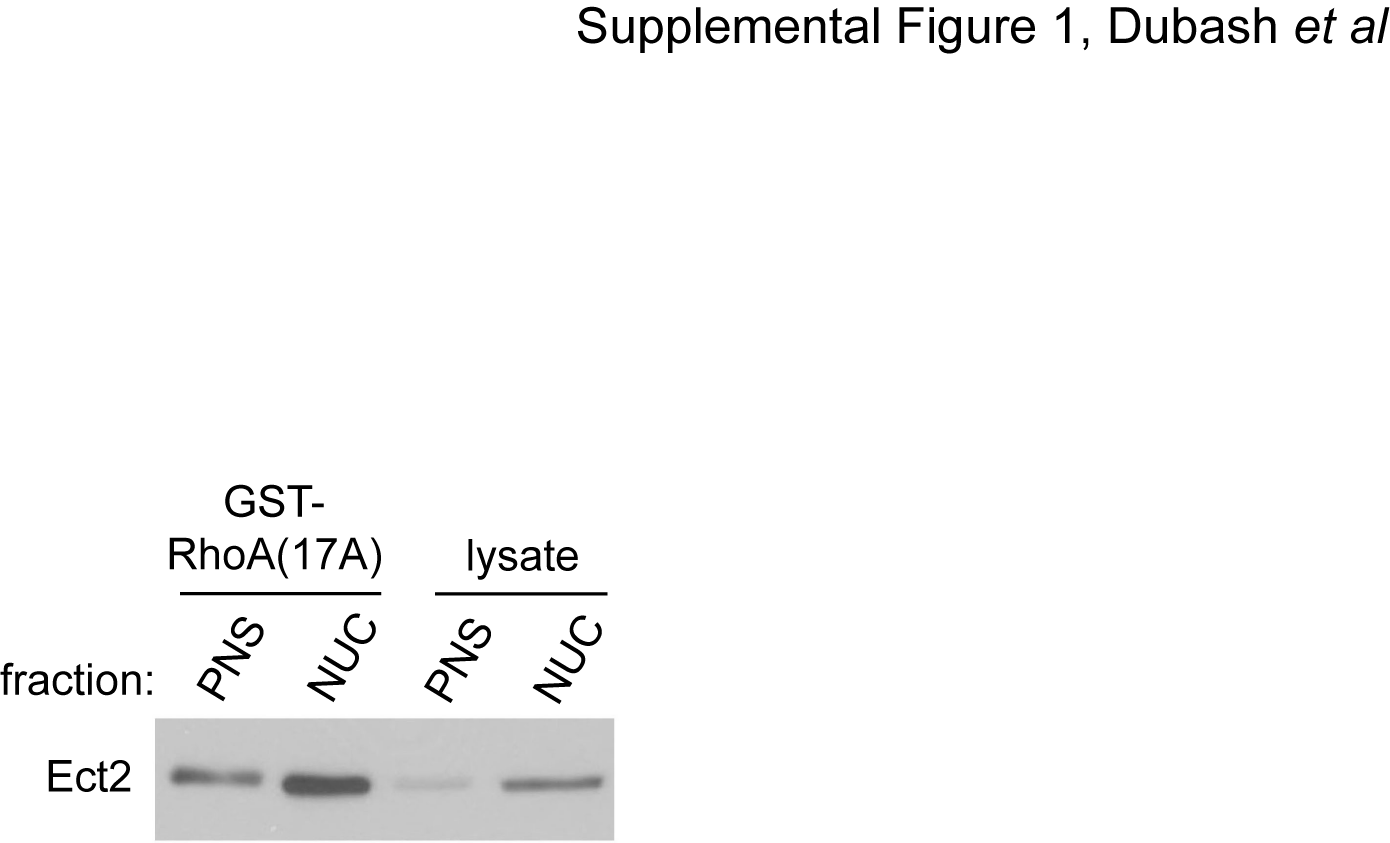

Supplement: Figure S1 — The RhoA GEF Ect2 is active in the nucleus of cells. Active GEF pulldowns with GST-RhoA(17A) were performed from PNS and nuclear fractions of HEK293 cells, and the samples blotted with antibodies for the GEF Ect2. (TIF) [file pone.0017380.s001.tif]

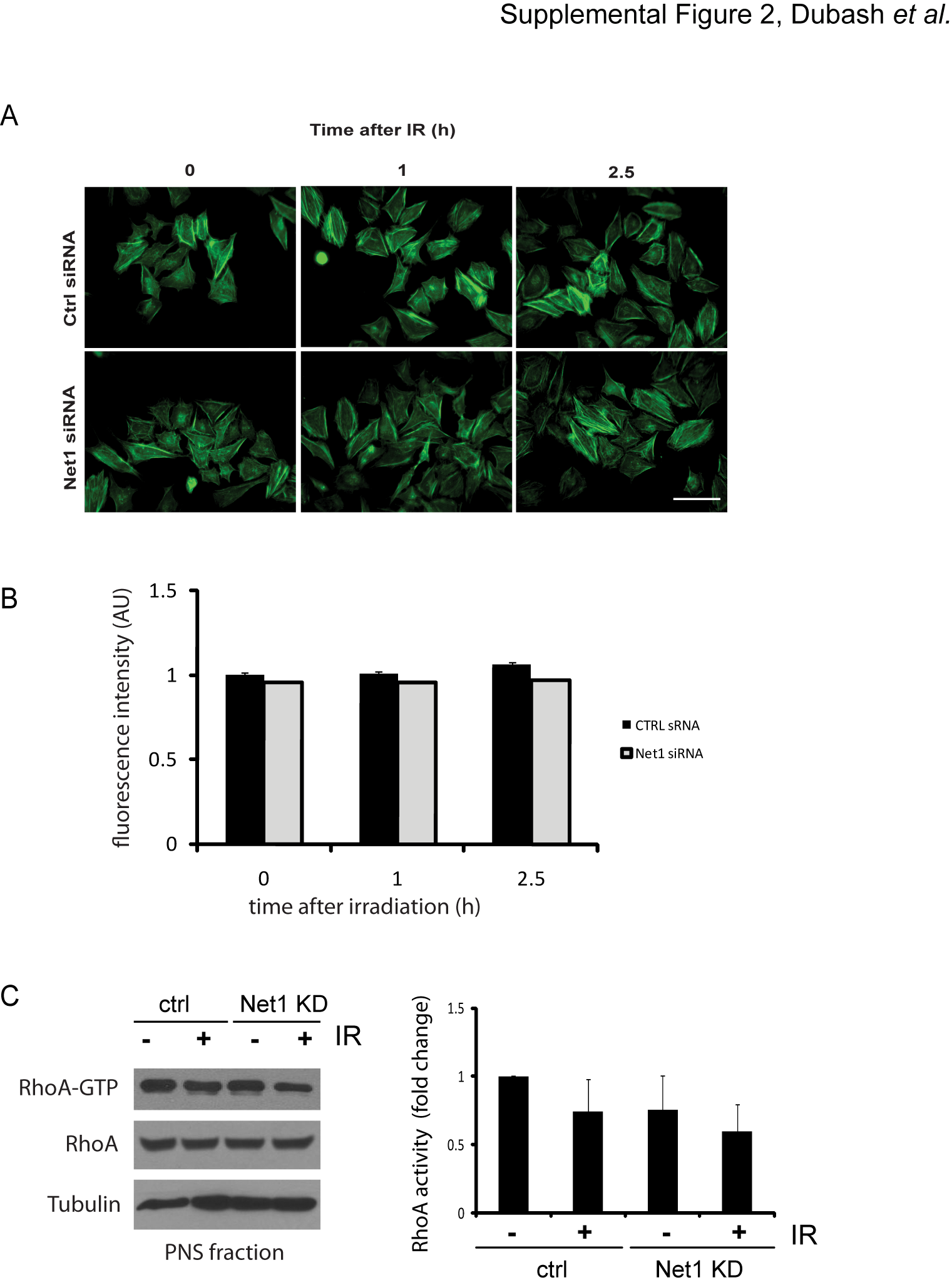

Supplement: Figure S2 — IR does not affect cytosolic RhoA signaling. (A) HeLa cells grown on coverslips were exposed to ionizing radiation (10 Gy), and fixed after incubation at 37°C for the indicated times. Cells were then stained with Alexa 488-Phalloidin to visualize F-actin. (B) Stress Fibers were quantified as described in Materials and Methods (n = 180 for each time point). Bar = 10µM. (C) HEK293 cells were transfected with control siRNA or Net1-specific siRNA. 72 hours post transfection, cells were either left untreated or exposed to ionizing radiation (10 Gy). After IR, cytosolic fractions were processed for RhoA activity assays and blotted for RhoA and Tubulin. This experiment was done simultaneously with the one shown in Figure 4E so the panel showing the efficiency of Net1 KD is shown there. Quantification of cytoplasmic (PNS) RhoA activity from three independent experiments (n = 3) is shown in the bar graph, as fold change over control cells. (TIF) [file pone.0017380.s002.tif]
